# The Prevalence and Awareness of Cardiometabolic Risk Factors in Southern Chinese Population with Coronary Artery Disease

**DOI:** 10.1155/2013/416192

**Published:** 2013-10-10

**Authors:** Xinrui Li, Yuan Zhang, Min Wang, Xiaofei Lv, Dongfang Su, Zhongxia Li, Ding Ding, Min Xia, Jian Qiu, Gang Hu, Wenhua Ling

**Affiliations:** ^1^Department of Nutrition, School of Public Health, Sun Yat-sen University (Northern Campus), 74 Zhongshan Road 2, Guangzhou, Guangdong 510080, China; ^2^Department of Cardiology, General Hospital of Guangzhou Military Command of People's Liberation Army, Number 111 Liuhua Road, Guangzhou, Guangdong 510010, China; ^3^Chronic Disease Epidemiology Laboratory, Pennington Biomedical Research Center, Baton Rouge, LA 70808, USA

## Abstract

*Background*. Cardiometabolic risk factors significantly accelerate the progression of coronary artery disease (CAD); however, whether CAD patients in South China are aware of the prevalence of these risk factors is not clear yet. *Methods*. The study consisted of 2312 in-admission CAD patients from 2008 to 2011 in South China. Disease history including hypertension, dyslipidemia, and diabetes was relied on patients' self-reported records. Physical and clinical examinations were tested to assess the real prevalence of the cardiometabolic risk factors. *Results*. 57.9% of CAD patients had more than 3 cardiometabolic risk factors in terms of the metabolic syndrome. The self-known and real prevalence of hypertension, diabetes, and dyslipidemia were 56.6%, 28.3%, and 25.1% and 91.3%, 40.9%, and 92.0%, respectively. The awareness rates were 64.4%, 66.3%, and 28.5% for hypertension, diabetes, and dyslipidemia. The prevalence of cardiometabolic risk factors was significantly different among gender and among disease status. *Conclusions*. Most CAD patients in South China had more than three cardiometabolic risk factors. However, the awareness rate of cardiometabolic diseases was low, especially for dyslipidemia. Strategies of routine physical examination programs are needed for the early detection and treatment of cardiometabolic risk factors in order to prevent CAD progression and prognosis.

## 1. Introduction

Coronary artery disease (CAD), one of the major causes of morbidity and mortality worldwide [1], had become a public health problem in China during the past few decades. Recently, age-standardized CAD mortality increased in China, in contrast to the declining trends in various developed countries [2]. The overall CAD mortality rate (per 100000 individuals per year) in China was projected to rise from 95.3 in 1999 to 103.4 in 2008 [3]. In 2008, crude morbidity rate of ischemic heart disease was 12.7% among Chinese urban residents [4].

Cardiometabolic risk factors, including obesity, elevated blood pressure, triglycerides, and plasma glucose, and reduced high density lipoprotein cholesterol, are strong contributors to CAD development. More than 80% of CAD patients have at least one of these risk factors [5], but the awareness rates of these risk diseases are very low among the Chinese population. The First China National Nutrition and Health Survey showed that only 24% of hypertension patients were aware of their condition [6]. The International Collaborative Study of Cardiovascular Disease in Asia demonstrated that, among those who had a high cholesterol level, the proportion of awareness was approximately 8% [7]. The China Heart Survey identified 87.4% undiagnosed impaired glucose regulation patients in 3513 participants hospitalized for CAD [8]. However, there are sparse data on prevalence and awareness of all the cardiometabolic risk factors in CAD population in developing countries. These risk factors affect how many CAD patients in South China and patients' awareness of these problems are not known. Determining the validated data of this question is important to guide the practice of clinical medicine and public health policies. In addition, physicians and patients can better understand the impact of preventing or modifying the specific risk factors on the risk of CAD progression.

The purpose of the present study is to investigate the prevalence and awareness of cardiometabolic diseases—hypertension, diabetes, and dyslipidemia—in South Chinese CAD patients. Moreover, the prevalence of metabolic syndrome is determined by physical examination and clinical tests to assess the real health status in this population.

## 2. Materials and Methods

### 2.1. Study Sample

Four superior specialties hospitals in Guangzhou, Guangdong, China, were chosen as study centres for the Guangdong Coronary Artery Disease Cohort. The study began in October 2008 and the recruitment period finished in December 2011. During this period, all in-admission patients who were diagnosed with CAD at the Department of Cardiology underwent eligibility screening. The inclusion criteria were (1) a history of or newly diagnosed CAD, (2) age ≥ 40 years at baseline survey, and (3) having stayed in Guangdong province at least 5 years. The exclusion criteria were (1) age < 40 or >85 years, (2) other cardiac origins (aortic valve stenosis or insufficiency, aortic dissection, acute pericarditis, rheumatic coronaritis, hypertrophic cardiomyopathy, syphilitic aortic regurgitation and cardioneurosis) and noncardiac (respiratory, gastrointestinal, or musculoskeletal) chest pain, (3) severe liver and/or kidney failure, and (4) a history of or newly diagnosed autoimmune disease or thyroid disorder. The study was approved by Sun Yat-sen University ethnic committee, and all participants or their designated relatives signed the informed consent.

### 2.2. Ascertainment of Coronary Artery Disease

The coronary artery disease cases were identified by using International Classification of Diseases (ICD-10) codes (I20-25). Every patient was diagnosed by cardiologists in the four hospitals according to the World Health Organization 1999/2000 guidelines [9, 10]. Eligible diseases were defined as the occurrence of at least two of the following situations: (1) typical chest pain with the property of oppression, dullness and constriction which occurred in the middle and upper part of sternum before, (2) ST-segment deviation of 0.05 mV or more and/or T wave inversion on first or histological electrocardiograph, (3) increased troponin and/or creatine kinase MB on initial blood tests, and/or at least one of the following situations: (1) measurable stenotic valve lesions of coronary arteries by coronary angiography and (2) history of percutaneous coronary intervention or coronary artery bypass grafting. Acute coronary syndrome (ACS) was defined as the occurrence of any of unstable angina pectoris, ST-segment elevation myocardial infarction, and non-ST-segment elevation myocardial infarction within 3 months. Severity of CAD was based on coronary angiography reports, characterized by coronary artery stenosis degree (categorized as not conduct, <50%, 50–74.9%, and ≥75%) and whether triple vessel disease or not. Treatment information of CAD included percutaneous coronary intervention (PCI) and coronary artery bypass graft (CABG).

### 2.3. Clinical Baseline Examinations

Patients' venous blood samples were collected in the morning after fasting for 12 hours. A standardized questionnaire on general information including gender, birth date and place, address, marriage status, education level, physical activities, smoking and alcohol drinking habits, and medication history and a validated food frequency questionnaire [11] were conducted through a face-to-face interview. Smoking was defined as at least one cigarette a day and lasting more than half a year. Smoking status was classified as never, past, or current. Alcohol drinking was defined as persons who drank any type of alcoholic beverages at least once a week [12] and lasting more than six months. Alcohol drinking status was divided into never, past, or current. Medication history of hypertension, diabetes, and dyslipidemia was documented if a diagnosis had been made prior to admission and/or under relevant drugs treatment for more than 2 weeks.

Clinical measurements, including height, weight, waist circumference (WC), blood pressure, blood lipid, and fasting plasma glucose (FPG), were measured by standardized methods in each hospital. Body mass index (BMI) was calculated as weight in kilograms divided by height in meters squared. Hypertension was defined as systolic blood pressure (SBP) ≥140 mmHg and/or diastolic blood pressure (DBP) ≥90 mmHg and/or having a hypertension history. Diabetes was defined as FPG level ≥7.0 mmol/L and/or having a diabetes history. Dyslipidemia was defined as any one of triglyceride (TG) ≥2.26 mmol/L, total cholesterol (TC) ≥6.22 mmol/L, low-density lipoprotein cholesterol (LDL-c) ≥4.14 mmol/L, and high-density lipoprotein cholesterol (HDL-c) < 1.04 mmol/L and/or having a dyslipidemia history. The metabolic syndrome was defined as the presence of at least 3 of the following 5 factors: obesity (WC ≥90 cm for male and ≥80 cm for female), elevated TG (≥1.7 mmol/L), reduced HDL-c (<1.0 mmol/L for male and <1.3 mmol/L for female), elevated blood pressure (≥130 mmHg for SBP and ≥85 mmHg for DBP), and elevated FPG (≥5.6 mmol/L) [13]. Since WC was only available in 1303 patients, BMI ≥25 kg/m^2^ [14] was also used to define obesity. 

### 2.4. Statistic Analysis

Continuous data were presented as means (SDs) in total group and means (SEs) in different gender or disease status groups. Categorical data were presented as percentages. The general linear model and chi-square test were used to analyze the differences in continuous variables and in categorical variables between men and women or between subjects with acute and stable coronary artery disease. Statistical significance was considered to be *P* < 0.05. All statistical analyses were performed with PASW for Windows, version 20.0 (IBM SPSS Inc, Chicago, IL, USA).

## 3. Results and Discussion

**Figure 1:**
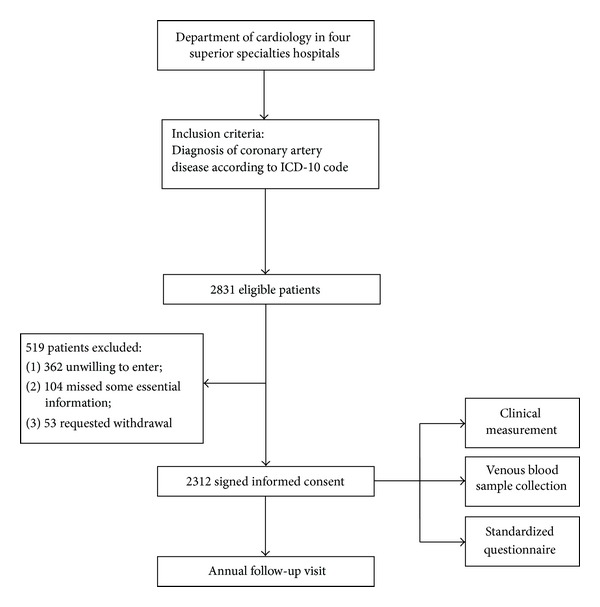
Recruitment period trail profile for Guangdong Coronary Artery Disease Cohort study.

A total of 2831 eligible patients were invited to the study and informed the studying procedures in detail. After excluding 362 subjects who were unwilling to sign informed consent, 104 missing some essential information, and 53 requesting withdrawal before discharge, 2312 subjects were included in the final analysis and would be followed in the future (Figure 1). 

Males comprised of 67.1% of the population in this study (Table 1). In general, male CAD patients were younger, had a higher education level, and were more often married, current smokers, alcohol drinkers, shorter CAD duration and with more severe disease status compared with female CAD patients. Male CAD patients had significantly higher prevalence of dyslipidemia and lower prevalence of central obesity, the metabolic syndrome, elevated TG (≥1.7 mmol/L), and reduced HDL-c (<1.0 mmol/L for male and <1.3 mmol/L for female) than female CAD patients. The awareness rates of hypertension, diabetes, and dyslipidemia were all significantly higher in female patients. 

About 60% of CAD patients were diagnosed with ACS at admission (Table 2). ACS patients were younger and with lower BMI and blood pressure, and they were more frequent smokers, and alcohol drinkers and with shorter CAD duration and more severe disease status than stable CAD patients. When compared with stable CAD patients, ACS patients had higher prevalence of hypertension, diabetes, dyslipidemia, elevated TG, and elevated FPG (≥5.6 mmol/L) but lower central obesity (WC > 85 cm for male and >80 cm for female). The awareness rates of hypertension and dyslipidemia were significantly lower in ACS patients than those in stable CAD patients. The difference between ACS and stable CAD in male patients was similar to the whole population, except that there were less physical activities and more metabolic syndrome in male ACS patients. However, among female patients, there were no statistical differences in cardiometabolic risk factors, diseases prevalence, and awareness between ACS and stable CAD patients.

The present study assessed the health status among 2312 inpatients with CAD in South China. The main finding was the high prevalence of cardiometabolic risk factors among patients with CAD including hypertension, diabetes, dyslipidemia, and the metabolic syndromes. More than half of the patients were aware of their hypertension or diabetes status, but few patients were aware of dyslipidemia. Compared to the male patients, female patients with CAD had higher cardiometabolic risk factors except for dyslipidemia. ACS patients had higher disease prevalence rates but lower awareness rate. 

Hypertension, diabetes, and dyslipidemia are labelled as conventional risk factors for their strength of evidence supporting role in the pathogenesis of CAD. It has been indicated that about 80–90% of CAD patients have at least one of the conventional risk factors [5]. In the present study, we collected the data from physical examination and laboratory tests which provided more convincing information on cardiometabolic risk factors. Our investigation found that the prevalence of hypertension, diabetes, and dyslipidemia among CAD patients was 91.3%, 40.9%, and 92.0%, respectively, and the unawareness rates of hypertension, diabetes, and dyslipidemia were 35.6%, 33.7%, and 71.5%.

High blood pressure is an important public-health challenge and a great contributor to CAD morbidity and mortality. A previous study had indicated that hypertension was a greater health burden in developing countries rather than developed ones [15]. The average blood pressure level among our subjects was similar to the report of the CAD patients in the 2007-2008 China National Diabetes and Metabolic Disorders Study [16], and the prevalence of hypertension accorded with the report from the study on Reduction of Atherothrombosis for Continued Health Registry (REACH registry) [17]. Our study showed that 64.4% of hypertension patients were aware of their hypertensive condition. The awareness rate was significantly higher than that of the general population in Guangdong [18] but still a bit lower than that of America adults [19]. 

Both the Euro heart survey and the China heart survey [8, 20] demonstrated that abnormal glucose regulation was around 60% in patients with CAD. Using oral glucose tolerance tests, Euro heart survey discovered 26.9% undiagnosed diabetes [21], and, in China heart survey, the proportion was 17.8% [8]. In concern, we identified 58.0% of patients with elevated FPG (≥5.6 mmol/L) and 20.2% newly diagnosed diabetes in total. It has long been known that people with diabetes have higher mortality rates and a 2–4 times greater risk for future coronary cardiovascular disease events compared with people without type II diabetes [22, 23]. Therefore, more effective clinical treatment strategies for diabetic CAD patients are needed in our population. 

The prevalence of dyslipidemia among CAD patients in Chinese multiprovince ACS study was 19.6% [24]; however, the dyslipidemia prevalence was 92.0% in our population, including 71.5% unawareness dyslipidemia. The national data included 7 geographic districts of China where the prevalence of dyslipidemia was lower in West and Middle China. Furthermore, the national study only considered TC and LDL-c to define dyslipidemia, while we defined dyslipidemia by using four biomarkers: TG, TC, LDL-c, and HDL-c. Therefore, the national average dyslipidemia prevalence was much lower than that in our study. An international randomized clinical trial [5] reported that the prevalence of dyslipidemia was 39.6% in women and 34.1% in men in the patients with CAD. But they might underestimate the true prevalence because they relied on patient self-report of risk factors. In the United States, the awareness rate of dyslipidemia was about 50.4% during 2005-2006 [25]. Unfortunately, the awareness rate was much lower in our population, because few patients accessed to regulate physical examination. Actually, high cholesterol is the biggest contribution to CAD incidence. Early awareness and proper treatment of dyslipidemia may lower 77% of overall deaths of CAD patients [2].

The metabolic syndrome is a complex index of cardiovascular risks. The present study revealed that female patients had higher prevalence of cardiometabolic diseases than males in terms of the metabolic syndrome and its individual components. This phenomenon could not be explained by the fact that female patients were 5 years older than male patients in our study, since CAD progression was usually several years later in women than in men [26]. It is well known that central obesity together with hyperglycemia is a powerful risk factor in women, which may attenuate the usual protection CAD women have [27]. Furthermore, in this study, there was higher prevalence of elevated TG and reduced HDL-c in female patients though the female had lower prevalence of dyslipidemia. This might indicate a better control of lipid dysfunction in male patients. The comparison between ACS and non-ACS patients also showed that the higher the prevalence of cardiometabolic diseases, the more severe ACS events. Therefore, great attention should be paid to female patients with the metabolic syndrome. 

The higher prevalence of several cardiometabolic risk factors in this study compared with other studies can be explained in several aspects. One potential reason is that the incidence of chronic disease increased rather rapidly over the past decade in China [6, 7, 16, 28, 29], which would influence the proportion of cardiometabolic diseases with CAD. Another explanation may be the detrimental changes in diet and lifestyle accompanying economic development and modernization. Traditional Chinese diet rich in cereal and vegetables is dramatically changing to western-style diet. More meat and dessert intake results in weight gain, higher blood lipid, and insulin resistance, which increases CAD risk [30]. Vehicle is a substitute for bicycle and walking leading to an inactive status, which may increase CAD mortality [31]. More noticeably, the average education levels were low in this population, with 61.4% attained less than 9 years education. Abundant studies report that education levels inversely associate with diseases risk and passively relate to disease awareness and treatment compliance [32]. In the present study, female patients usually obtained much less education, and this might partly explain the higher prevalence of metabolic risk factors in female patients. 

Developing countries have entered the so-called “epidemiological transition,” and noncommunicable diseases mortality will account for more than 70% of all-cause deaths in 2020 [33]. The current strategies aiming at CAD prevention include mass education campaigns, diet and lifestyle interventions, and pharmacological treatment. However, these strategies are ineffective at reducing the risk factors of CAD. Most cases with unaware dyslipidemia in the present study indicate a low routine physical examination rate to the patients or general populations. Therefore, more efforts should be made in controlling complications and implementing secondary prevention for CAD patients in addition to the education on dietary pattern and lifestyle. 

There were several strengths in our study, including large sample size and cardiometabolic diseases confirmation by laboratory tests. In addition, biologic samples were collected from every cohort member, which facilitated the testing of endogenous exposures and genetic factors for etiology of CAD. One limitation of our study was that some severe cases and terminally ill patients were excluded in the cohort because they could not sign the informed consent, which limited our ability to research the distinguishing features for these patients. However, investigators tried to communicate with their family members to ensure a sufficiently small refusal rate (less than 10%). Another limitation was that WC was only available in 1303 participants for the metabolic syndrome definition, but our further study did not find any difference in baseline characteristics between patients with and without WC data.

## 4. Conclusion

Our data reported high prevalence of cardiometabolic risk factors in a South Chinese population with CAD and showed low disease awareness rates among the patients. There is a large variation in CAD prevalence between northern and southern Chinese [2], and our study attenuates the paucity of high-quality data about CAD in South China. Further study of this CAD population will provide a valuable opportunity to evaluate many important etiologic hypotheses for CAD which may be conducted only in this Chinese cohort.

## Figures and Tables

**Table 1:** Patients demographic stratified by gender.

Variable	Total (*n* = 2312)	Male (*n* = 1553)	Female (*n* = 759)	*P*
Age (years)	63.7 ± 11.4	62.0 ± 0.3	67.3 ± 0.4	<0.001
Body mass index (kg/m^2^)	23.9 ± 3.3	23.8 ± 0.1	24.1 ± 0.1	0.129
Systolic blood pressure (mmHg)	132 ± 23	131 ± 1	134 ± 1	0.001
Diastolic blood pressure (mmHg)	77 ± 13	77 ± 1	77 ± 1	0.694
Total cholesterol (mmol/L)	4.63 ± 1.12	4.48 ± 0.03	4.94 ± 0.04	<0.001
Low-density lipoprotein cholesterol (mmol/L)	2.91 ± 1.25	2.84 ± 0.03	3.06 ± 0.05	<0.001
High-density lipoprotein cholesterol (mmol/L)	1.06 ± 0.29	1.01 ± 0.01	1.16 ± 0.01	<0.001
Triglycerides (mmol/L)	1.81 ± 1.31	1.76 ± 0.03	1.91 ± 0.05	0.009
Lysophosphatidic acid (g/L)	0.36 (0.22, 0.76)	0.38 (0.22, 0.77)	0.35 (0.22, 0.71)	0.149
Apolipoprotein A (g/L)	1.10 ± 0.28	1.05 ± 0.01	1.19 ± 0.01	<0.001
Apolipoprotein B (g/L)	0.78 ± 0.28	0.76 ± 0.01	0.83 ± 0.01	<0.001
Fasting glucose (mmol/L)	6.38 ± 2.62	6.26 ± 0.07	6.63 ± 0.10	0.002
Uric acid (umol/L)	376 ± 113	390 ± 3	348 ± 4	<0.001
Duration of coronary artery disease (years)				
First diagnosed patients (*n* = 1223)	—	—	—	
History diagnosed patients (*n* = 1089)	3.00 (1.00, 8.00)	2.29 (0.91, 6.66)	4.45 (1.12, 10.0)	<0.001
Marriage (%)	89.9	93.8	81.6	<0.001
Years of education (%)				<0.001
≤9	61.4	57.2	69.6	
10–12	20.0	21.8	16.8	
≥13	18.6	21.0	13.6	
Physical activities (%)				0.101
None	35.9	38.1	31.4	
≤30 min	20.9	20.1	22.6	
>30 min	43.2	41.8	46.0	
Smoking (%)				<0.001
Never	59.0	41.3	95.4	
Past	8.0	10.9	2.0	
Current	32.9	47.7	2.6	
Alcohol drinking (%)				<0.001
Never	77.9	67.6	98.2	
Past	6.9	10.2	0.4	
Current	15.2	22.2	1.4	
Type of coronary artery disease (%)				<0.001
Stable status	41.0	36.5	50.2	
Unstable angina pectoris	35.1	30.4	36.1	
Myocardial infarction	23.9	33.1	13.7	
Coronary artery stenosis degree of CAG				<0.001
Not conduct	34.9	28.0	49.0	
<50%	12.1	10.7	15.0	
50–74.9%	7.9	8.0	7.8	
≥75%	45.1	53.3	28.2	
Triple vessel disease (%) (*n* = 1181 with CAG reports)	51.1	53.5	43.7	<0.001
Treatment of coronary disease (%)				
Percutaneous coronary intervention	50.6	59.1	34.2	<0.001
Coronary artery bypass graft	2.3	2.8	1.2	0.018
History of diseases (%)				
Hypertension	56.6	52.8	63.9	<0.001
Diabetes mellitus	28.3	25.7	33.6	<0.001
Dyslipidemia	25.1	23.8	27.6	0.046
Current prevalence of diseases (%)				
Hypertension (≥140/90 mmHg for SBP/DBP or drug use)	91.3	91.4	91.0	0.747
Diabetes mellitus (≥7.0 mmol/L for FPG or drug use)	40.9	39.6	43.3	0.111
Dyslipidemia*	92.0	93.7	88.9	<0.001
Awareness rate of disease (%)				
Hypertension	64.4	60.7	71.7	<0.001
Diabetes mellitus	66.3	63.3	71.6	0.011
Dyslipidemia	28.5	26.6	32.7	0.005
The metabolic syndrome and its individual components (%)				
Defined by BMI ≥25 kg/m^2^	57.9	54.3	65.0	<0.001
Defined by BMI ≥25 kg/m^2^ (*n* = 1303 with waist circumference)	58.5	54.8	67.2	<0.001
Defined by waist circumference (*n* = 1303)	66.9	61.2	80.2	<0.001
Obesity (BMI ≥25 kg/m^2^)	34.1	34.5	33.5	0.647
Obesity (waist circumference >85/80 cm for male/female)	62.5	53.8	83.2	<0.001
Triglycerides (≥1.70 mmol/L)	38.8	36.9	42.5	0.010
High-density lipoprotein cholesterol (<1.0/1.3 mmol/L for male/female)	56.5	50.3	69.1	<0.001
High blood pressure (≥130/85 mmHg for SBP/DBP)	93.7	93.7	93.6	0.922
Fasting plasma glucose (≥5.6 mmol/L)	58.0	56.6	60.9	0.055

Only 1303 but not all study samples measured waist circumference. CAG: coronary angiography.

*Dyslipidemia was defined as any one of triglyceride ≥2.26 mmol/L, total cholesterol ≥6.22 mmol/L, low-density lipoprotein cholesterol ≥4.14 mmol/L, high-density lipoprotein cholesterol <1.04 mmol/L, and/or using cholesterol-lowering medicines during the last 2 weeks.

**Table 2:** Patients demographic stratified by disease status.

Variable	Male			Female			Total		*P* value
	ACS (*n* = 986)	Others (*n* = 567)	*P* value	ACS (*n* = 378)	Others (*n* = 381)	*P* value	ACS (*n* = 1364)	Others (*n* = 948)	
Age (years)	61.2 ± 0.4	63.3 ± 0.5	0.001	67.1 ± 0.5	67.1 ± 0.5	0.941	63.1 ± 0.3	64.5 ± 0.4	0.004
Body mass index (kg/m^2^)	23.8 ± 0.1	24.2 ± 0.1	0.026	23.8 ± 0.2	24.1 ± 0.2	0.232	23.8 ± 0.1	24.1 ± 0.1	0.014
Systolic blood pressure (mmHg)	130 ± 1	132 ± 1	0.021	134 ± 1	136 ± 1	0.325	131 ± 1	133 ± 1	0.017
Diastolic blood pressure (mmHg)	76 ± 1	77 ± 1	0.051	76 ± 1	76 ± 1	0.558	76 ± 1	77 ± 1	0.052
Total cholesterol (mmol/L)	4.52 ± 0.04	4.45 ± 0.05	0.306	4.94 ± 0.06	4.87 ± 0.06	0.428	4.66 ± 0.03	4.59 ± 0.04	0.168
Low-density lipoprotein cholesterol (mmol/L)	2.89 ± 0.04	2.77 ± 0.06	0.097	3.06 ± 0.05	2.99 ± 0.05	0.337	2.95 ± 0.03	2.84 ± 0.04	0.050
High-density lipoprotein cholesterol (mmol/L)	0.99 ± 0.01	1.06 ± 0.01	<0.001	1.15 ± 0.02	1.18 ± 0.02	0.223	1.04 ± 0.01	1.10 ± 0.01	<0.001
Triglycerides (mmol/L)	1.82 ± 0.04	1.74 ± 0.06	0.310	1.83 ± 0.06	1.86 ± 0.06	0.728	1.83 ± 0.04	1.78 ± 0.05	0.432
Lysophosphatidic acid (g/L)	0.39 (0.23, 0.88)	0.34 (0.21, 0.65)	0.005	0.39 (0.22, 0.86)	0.33 (0.20, 0.58)	0.023	0.39 (0.23, 0.87)	0.34 (0.21, 0.61)	<0.001
Apolipoprotein A (g/L)	1.02 ± 0.01	1.11 ± 0.01	<0.001	1.17 ± 0.01	1.24 ± 0.02	<0.001	1.07 ± 0.01	1.15 ± 0.01	<0.001
Apolipoprotein B (g/L)	0.78 ± 0.01	0.73 ± 0.01	<0.001	0.85 ± 0.02	0.79 ± 0.02	0.018	0.81 ± 0.01	0.75 ± 0.01	<0.001
Fasting glucose (mmol/L)	6.45 ± 0.08	5.97 ± 0.11	<0.001	7.07 ± 0.16	6.09 ± 0.16	<0.001	6.64 ± 0.07	5.98 ± 0.09	<0.001
Uric acid (umol/L)	385±4	404 ± 5	0.002	342 ± 6	358 ± 6	0.041	371 ± 3	388 ± 4	<0.001
Duration of coronary artery disease (years)									
First diagnosed patients (*n* = 1223)	—	—		—	—		—	—	
History diagnosed patients (*n* = 1089)	2.00 (0.77, 6.31)	2.42 (1.00, 6.99)	0.031	3.21 (0.94, 9.78)	5.07 (2.00, 10.5)	0.002	2.43 (0.79, 7.15)	3.67 (1.17, 8.96)	<0.001
Marriage (%)	94.5	92.7	0.329	79.5	83.7	0.316	90.5	89.1	0.454
Years of education (%)			0.595			0.207			0.271
≤9	57.9	56.4		72.3	66.8		62.1	60.5	
10–12	20.7	23.1		16.5	17.0		19.5	20.7	
≥13	21.4	20.5		11.2	16.2		18.4	18.8	
Physical activities (%)			0.030			0.445			0.066
None	41.7	31.9		29.8	33.2		38.3	32.4	
≤30 min	18.2	23.4		22.6	22.6		19.5	23.1	
>30 min	40.1	44.7		47.6	44.2		42.2	44.5	
Smoking (%)			<0.001			<0.001			<0.001
Never	37.5	48.3		94.7	96.1		53.4	67.6	
Past	8.9	14.7		1.6	2.5		6.9	9.8	
Current	53.6	37.0		3.7	1.4		39.7	22.7	
Alcohol drinking (%)			0.094			0.021			0.006
Never	68.7	65.7		98.6	97.7		77.4	78.6	
Past	9.5	11.4		0.0	0.8		6.8	7.1	
Current	21.8	22.8		1.4	1.5		15.8	14.3	
Coronary artery stenosis degree of CAG			<0.001			<0.001			<0.001
Not conduct	19.6	43.4		39.9	58.5		25.2	49.5	
<50%	6.5	18.4		7.7	22.5		6.9	20.1	
50–74.9%	7.3	9.3		8.5	7.2		7.6	8.4	
≥75%	66.6	28.9		43.9	11.8		60.3	22.0	
Triple vessel disease (%) (*n* = 1181 with coronary angiography)	58.6	39.0	<0.001	54.8	25.4	<0.001	57.8	34.8	<0.001
Treatment of coronary disease (%)									
Percutaneous coronary intervention	71.9	37.9	<0.001	53.6	15.2	<0.001	66.5	28.1	<0.001
Coronary artery bypass graft	2.6	3.3	0.470	1.5	0.9	0.464	2.3	2.3	0.972
Hypertension	49.3	59.1	0.001	64.0	63.8	0.956	53.6	61.1	0.001
Diabetes mellitus	26.8	23.7	0.181	38.5	28.5	0.003	30.1	25.6	0.021
Dyslipidemia	23.9	23.7	0.918	26.0	29.3	0.305	24.5	26.0	0.423
Current prevalence of diseases (%)									
Hypertension (≥140/90 mmHg for SBP/DBP or drug use)	92.8	88.9	0.015	92.9	89.0	0.075	92.9	89.0	0.002
Diabetes mellitus (≥7.0 mmol/L for FPG or drug use)	42.1	35.1	0.014	49.3	37.1	0.001	44.2	36.0	<0.001
Dyslipidemia*	95.8	89.9	<0.001	91.2	86.6	0.057	94.4	88.5	<0.001
Awareness rate of disease (%)									
Hypertension	56.9	67.5	<0.001	70.7	72.8	0.542	60.9	69.7	<0.001
Diabetes mellitus	60.8	68.9	0.060	71.6	71.6	0.993	64.2	70.1	0.071
Dyslipidemia	25.2	29.3	0.102	29.6	35.9	0.090	26.4	31.9	0.007
The metabolic syndrome and its individual components (%)									
Metabolic syndrome (BMI ≥25 kg/m^2^)	56.5	50.0	0.016	65.8	64.2	0.640	59.1	55.9	0.129
Metabolic syndrome (BMI ≥25 kg/m^2^, *n* = 1303 with waist circumference)	56.3	51.9	0.227	66.8	67.6	0.880	58.9	58.0	0.776
Metabolic syndrome (waist circumference, *n* = 1303)	62.7	58.2	0.211	81.3	79.1	0.602	67.2	66.4	0.770
Obesity (BMI ≥25 kg/m^2^)	33.2	36.8	0.154	32.9	34.1	0.719	33.1	35.7	0.196
Obesity (waist circumference >85/80 cm for male/female)	52.2	56.7	0.191	82.1	84.2	0.585	59.4	67.1	0.005
Triglycerides (≥1.70 mmol/L)	39.8	31.3	0.001	43.3	41.7	0.645	40.8	35.5	0.012
High-density lipoprotein cholesterol (<1.0/1.3 mmol/L for male/female)	53.8	43.4	<0.001	69.0	69.2	0.955	58.0	54.0	0.060
High blood pressure (≥130/85 mmHg for SBP/DBP)	94.5	92.3	0.118	94.0	93.2	0.645	94.4	92.7	0.128
Fasting plasma glucose (≥5.6 mmol/L)	59.2	51.7	0.005	66.8	54.6	0.001	61.3	52.9	<0.001

Only 1303 but not all study samples measured waist circumference. CAG: coronary angiography.

*Dyslipidemia was defined as any one of triglyceride ≥2.26 mmol/L, total cholesterol ≥6.22 mmol/L, low-density lipoprotein cholesterol ≥4.14 mmol/L, and high-density lipoprotein cholesterol <1.04 mmol/L and/or using cholesterol-lowering medicines during the last 2 weeks.
